# Development and Characterization of Amorphous Thermoplastic Matrix Graphene Nanocomposites

**DOI:** 10.3390/ma5101972

**Published:** 2012-10-22

**Authors:** Antonio Greco, Alessia Timo, Alfonso Maffezzoli

**Affiliations:** Department of Innovation Engineering, University of Salento, Via per Arnesano, Lecce 73100, Italy; E-Mails: alessiatimo@hotmail.it (A.T.); alfonso.maffezzoli@unisalento.it (A.M.)

**Keywords:** graphene, nanocomposite, thermoplastic matrix

## Abstract

The aim of the present work is the development of amorphous thermoplastic matrix nanocomposites based on graphite nanoparticles. Different types of graphite were used, including unmodified graphite, graphene nanoplatelets and graphite intercalation compounds. Graphite intercalation compounds were subjected to thermal treatment to attain exfoliation of the nanofiller. The exfoliation process was studied by means of thermal analysis. The nanofillers and nanocomposites were characterized by means of X-ray Diffraction (XRD) and Scanning Electron Microscope (SEM) analysis. The nanocomposites were further characterized by means of mechanical and dielectric analysis. The flammability of the nanocomposites was also analyzed. Results obtained indicate that addition of the nanofiller allows improving the proprieties of the amorphous thermoplastic matrix. The effect of the degree of dispersion of the nanofiller is particularly relevant for the dielectric properties of the nanocomposites, whereas no direct correlation between degree of dispersion and mechanical properties can be observed.

## 1. Introduction

Polymer nanocomposites are a class of materials for which the addition of small amounts of inorganic phase, dispersed at nano-scale level, allows obtaining a significant improvement of properties compared to the base polymer. Both thermoplastic [1,2,3,4,5] as well as thermoset [6] based clay nanocomposites have been widely studied, showing that in most cases the improvement of properties [7,8] is counterbalanced by negative effects, as for example an increase in viscosity [9,10], a modification of the characteristic transition temperatures [11,12,13,14], a loss of transparency [15,16], and, in photoactivated systems, a decrease of the polymerization kinetics [17,18,19]. For this reason, academic research focuses on alternative materials for the production of polymer nanocomposites. These include addition of organic fillers (hyperbranched polymers) [20,21,22] or the use of different types of inorganic nanofillers [23,24,25,26]. 

Among the inorganic nanofillers, graphene has attracted great attention in recent years due to its exceptional thermal, mechanical, and electrical properties [27]. One of the most promising applications of this material is in polymer nanocomposites. In fact, due to the high aspect ratios [28], small amounts of graphene are sufficient to achieve percolation and network formation within a polymer matrix [29]. Therefore, significant property improvement can be achieved, with respect to electrical conductivity, barrier resistance, stiffness, abrasion resistance and fire retardancy at very low carbon content [30].

Scalable approaches to Graphene Nano Platelets (GNPs) and graphene-based materials (few-layer platelets or monolayer carbon sheets with heteroatoms and topological defects) primarily utilize Graphite Intercalation Compounds (GIC) or Graphene Oxide (GO) as the precursor material, respectively. Most exfoliated graphite fillers are derived from GICs, which are compounds of graphite with atoms or molecules (such as alkali metals or mineral acids) intercalated between the carbon layers [31]. The intercalation of graphite increases its interlayer spacing, weakening the interlayer interactions and facilitating the exfoliation of the GIC by mechanical or thermal methods [32]. Varying structural arrangements of the intercalant are possible, such as alternating layers of graphene and intercalant (referred to as first stage GICs), as well as multiple (two to five) adjacent graphene layers between intercalant layers (higher-stage GICs). It is the former arrangement, however, which is preferred for the complete exfoliation of these materials into monolayer platelets [33]. Intercalation of graphite by a mixture of sulfuric and nitric acid produces a higher-stage GIC that can be exfoliated by rapid heating or microwave treatment of the dried down powder, producing a material commonly referred to as expanded graphite (EG) [34]. EG retains a layered structure but has a slightly increased interlayer spacing relative to graphite, consisting of thin platelets (30–80 nm) which are loosely stacked. 

Solvent-based exfoliation and thermal exfoliation techniques have emerged as two preferred routes for this step. In the former route, the hydrophilic nature and increased interlayer spacing of GIC facilitates direct exfoliation into water assisted by mechanical exfoliation, such as ultrasonication and/or mechanical stirring [35]. GIC can also be exfoliated and reduced by rapid heating up to 400 °C at high rates such as 2000 °C/min [36]. The rapid heating is believed to cause various small molecule species (e.g., CO, CO_2_, water) to evolve and internal pressure to increase, forcing the sheets apart and yielding a dry, high-surface area material with a low bulk density.

Despite the great interest in graphene nanocomposites, most of the work cited in the literature deals with thermoset nanocomposites [37]. Only recently, thermoplastic nanocomposites have been prepared by melt mixing [38,39,40,41] or *in situ* polymerization, using GO itself as catalyst [42,43,44,45]. 

On the other hand, thermoplastic matrix composites and nanocomposites are finding increasing interest in both academic and industrial research, mainly due to the advantages of thermoplastics in terms of manufacturing costs, impact resistance, and environmental compatibility [46]. Analysis of the effect of nanofillers on thermoplastic matrix is rather complex, due to the influence of the nanofiller on the crystallization behavior of the matrix. Therefore, any observed modification of property can be assigned to either the addition of the nanofiller or to the modification of the crystalline structure of the matrix induced by the nanofiller itself. 

Therefore, this work is aimed at studying the applicability of graphite nanoparticles for the production of amorphous poly-ethylene-terephthalate (PETg) nanocomposites. Different carbon-based nanofillers were characterized, with particular focus on their morphology and response to thermal shocking and ultrasonic and mechanical stirring. PETg nanocomposites were produced by melt mixing, and the resultant mechanical and thermal properties were highlighted. 

## 2. Materials and Methods

The thermoplastic matrix used was an amorphous PET copolymer (PETg) “Eastar Copolyester 6763”, produced by Eastman. The graphite intercalation compound (GIC) used is produced by Anthracite Industries Inc. (Asbury Carbons) under the tradename 3772, and is characterized by a sulfur content of 3.5% and an expansion ratio of 290. Expanded graphite (GICe) was obtained by placing the GIC into an oven at 700 °C and holding for about 2 min. GICe was further sonicated to produce GICes. The unmodified graphite used was purchased from Sigma-Aldrich. Graphene nanoplatelets (GNP) were purchased from Cheap Tubes. Nanocomposites were produced by melt mixing in a Haake Rheocord mixer at 210 °C for 30 min with a constant amount (1% by weight) of nanofiller. 

Samples for mechanical and flame resistance characterization were obtained by preheating the materials in powder form (less than 500 μm diameter) up to 230 °C and then compression molding under 20 tons with cold (50 °C) plates in a Campana press.

Flexural tests were performed on a Lloyd LR 5K dynamometer, on 2 × 10 × 32 mm samples, using a cross head speed of 2 mm/min.

Thermo Gravimetric Analyzer (TGA) analysis was performed on GIC powders using a Netszch DTA/TGA thermal analyzer, heating 3 mg samples between 25 and 1000 °C at 10 °C/min in air atmosphere.

Themomechanical Analysis (TMA) was performed on GIC powders heating 3 mg samples between 25 and 450 °C at different heating rates and using different compression forces on the sample. Details on the the TMA experiment for powder characterization are reported elsewhere [5,47,48,49].

Scanning Electron Microscope (SEM) analysis was performed on a Zeiss EVO 40 microscope, equipped with Energy-Dispersive X-ray Spectroscopy (EDS) analysis. 

X-ray analysis was performed on both nanofiller powders and nanocomposites, within the range of 2θ = 1°–10°, using a Wide angle X-ray Diffractometer, RIGAKU Ultima +.

## 3. Results and Discussion

### 3.1. Characterization of Graphite Nanofillers 

**Figure 1:**
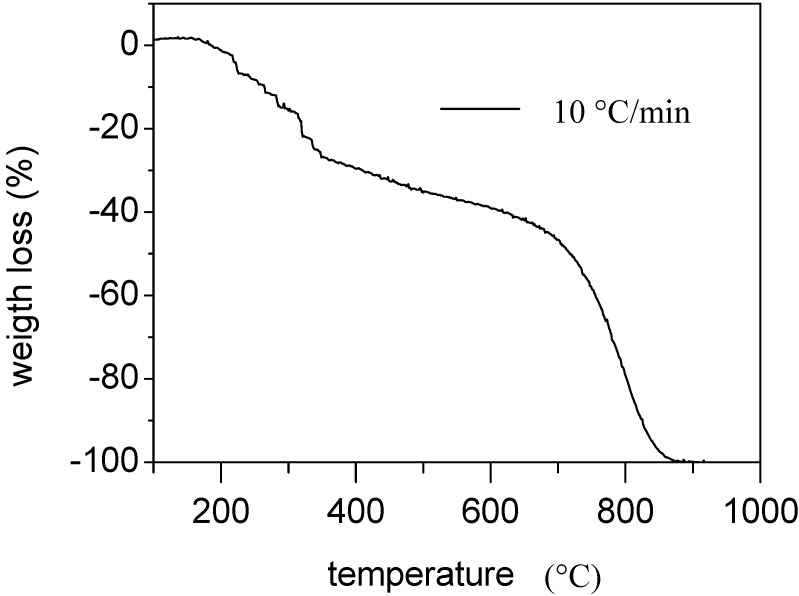
Thermo Gravimetric Analyzer (TGA) analysis of Graphite Intercalation Compounds (GIC).

The TGA analysis results for the GIC sample are reported in Figure 1, showing the presence of a double degradation step. The first step, with an onset temperature of 170 °C and an endset of about 400 °C, involves a weight loss of about 21%, which is due to the decomposition of the intercalating agent. The second step, with an onset of about 710 °C, is due to the thermal decomposition of carbon. 

**Figure 2:**
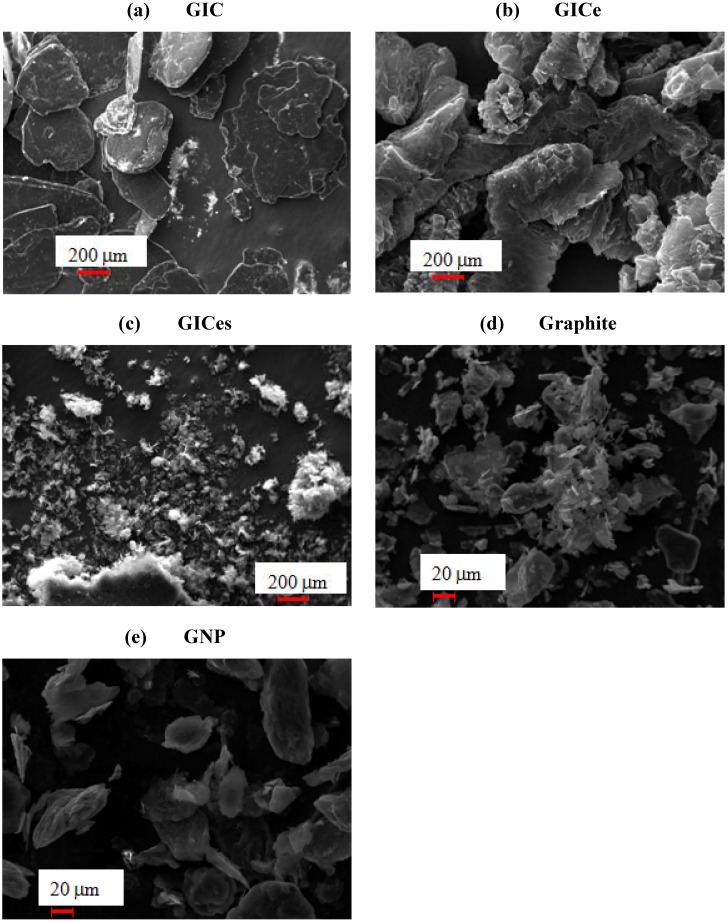
Scanning Electron Microscope (SEM) micrographs of graphite nanofillers.

SEM micrographs of expandable graphite before thermal treatment (sample GIC) are reported in Figure 2a, showing a structure characterized by an aggregate of lamellae. Each aggregate is disk-shaped, with an average diameter about 400–600 μm and a thickness about 50 μm [50]. After thermal treatment, the structure of the GIC is significantly modified, as observed in Figure 2b, characterized by the formation of a worm-like structure. The increase of the size of the lamellae, due to the decomposition of the intercalant gas, is well evidenced. The thickness of expanded lamellar stacks is about 300 times the initial thickness of lamellar stacks, whereas the diameter is not changed compared to original GIC. The SEM analysis performed on the sample GICes is reported in Figure 2c, where the presence of sub-micrometric lamellar aggregates is evidenced. A SEM micrograph of graphite is reported in Figure 2d, showing a structure very similar to that of GIC, although characterized by aggregates much smaller than those observed in Figure 2a for GIC. Finally, the SEM micrograph of GNP is reported in Figure 2e, showing that the structure is mainly constituted by clusters of lamellae, very similar to that observed in Figure 2c for GICes sample. 

EDS analysis reveals that the sulfur content of GIC before thermal treatment is about 3.88% by weight (corresponding to 1.53% molar), whereas the oxygen amount is 9.13% by weight (corresponding to 7.20% molar). The carbon amount is 87%. The amount of sulfur is reduced to 0.18% by weight (0.07% molar) after thermal treatment, whereas the oxygen amount is reduced to 4.44% by weight (3.38% molar). This indicates that the expansion of the GIC is due to the evaporation of some sulfur compound intercalated between the graphite lamellae. The presence of some residual sulfur in the GICes sample is in accordance with the TGA analysis results. In fact, the maximum temperature attained by the material during thermal treatment in the oven is about 350 °C, which is lower than the endset temperature of dissociation measured by TGA. This is responsible for the presence of some residual intercalating agent in the GICes sample. 

**Figure 3:**
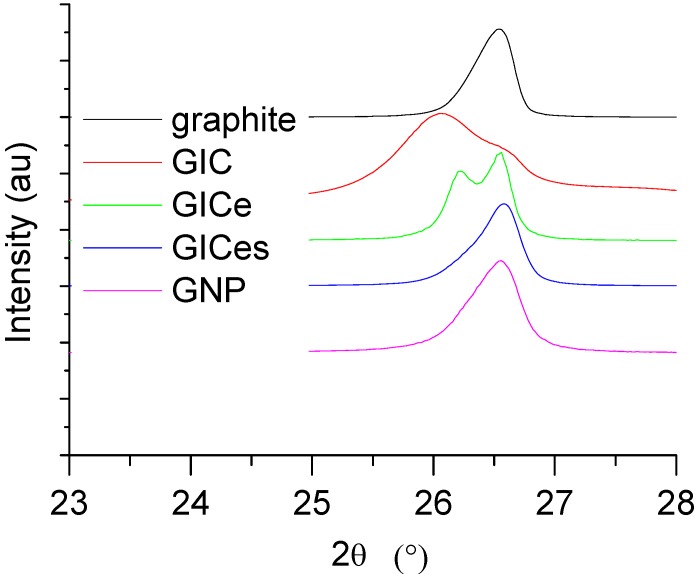
X-ray Diffraction (XRD) traces of graphite samples.

A comparison of X-ray Diffraction (XRD) traces for the different graphite samples is reported in Figure 3. The samples graphite and GNP are characterized by a single peak at 2θ = 26.55°, which is characteristic of non-intercalated graphite lamellae. 

The GIC compound before thermal treatment is characterized by the presence of two peaks, the first one occurring at 2θ = 26.05° and the second one at 2θ = 26.55°, which, according to the Bragg law, corresponds to d = 3.42 A and d = 3.35 A. The peak at 2θ = 26.55° corresponds to the peak of graphite and is therefore due to the presence of non-intercalated graphite lamellae, whereas the peak at 2θ = 26.05° is representative of the intercalated lamellae. After thermal treatment the relative intensity of the two peaks is inverted. In fact, for sample GICe, the peak at 2θ = 26.55° is higher than the peak at lower angles, which is shifted at 2θ = 26.2°. During thermal treatment, decomposition of the intercalation compound involves separation of the previously intercalated lamellae. Therefore, the amount of material characterized by a spacing corresponding to 2θ = 26.05° decreases. However, the presence of the peak at 2θ = 26.05°, even after thermal treatment, confirms that some of the intercalating agent is still present inside the galleries due to the fact that the maximum temperature attained during thermal treatment is lower than the endset of dissociation of the expanding compound. In fact, after sonication, the peak at d = 3.42 A vanishes, which indicates that the intercalating agent is removed by the lamellar galleries, due to solubilization in the sonicating liquid. The results found by XRD analysis are in very good agreement with similar systems studied by other authors [51], showing the presence of additional peaks in GIC before thermal treatment, which disappear after thermal treatment leading to complete dissociation of the expanding compound.

**Figure 4:**
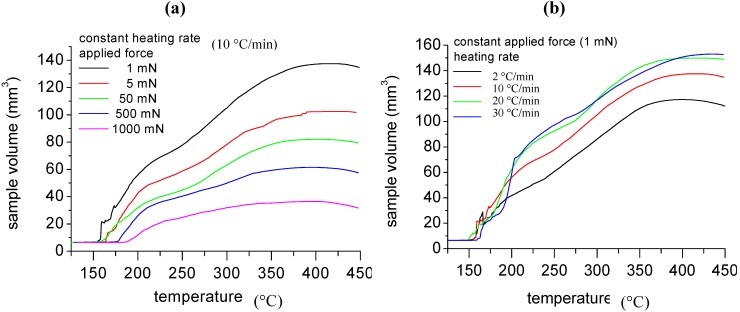
Themomechanical Analysis (TMA) of GIC (**a**) at different compression forces and (**b**) at different heating rates.

TMA analysis results for GIC tested at a constant heating rate (10 °C/min) under different compression forces are reported in Figure 4a. The data reported reveal that the expansion of GIC begins at about 160 °C, which roughly corresponds to the onset temperature of dissociation of the intercalating agent revealed by TGA analysis. The expansion proceeds by two steps and is almost completed at 400 °C. Increasing the applied pressure involves a shift of the expansion curves at higher temperatures and a reduction of the expansion ratio, defined as the ratio between the final volume of the material and its initial mass, as evidenced by the data reported in Table 1. 

The TMA curves obtained under a constant applied force of 1mN and at different heating rates are reported in Figure 4b. As it can be observed, increasing the heating rate does not involve any significant modification of the onset temperature, but has a dramatic influence on the expansion ratio of GIC. Increasing the heating rate involves an increase of the expansion ratio, as also observed in Table 2.

**Table 1:** Expansion ratio at different compression forces.

Compression force (mN)	RE (mm^3^/g)
1	69
5	51
50	41
500	31
1000	19

**Table 2:** Expansion ratio at different compression forces.

Heating rate (°C/min)	RE (mm^3^/g)
2	59
5	61
10	69
20	75
30	77

The expansion ratio values calculated by TMA analysis are much lower than those obtained by heating the GIC in the oven. In fact, during thermal treatment in the oven, the applied force is null (free expansion) and the heating rate can be estimated to be much higher (after 30 s the measured temperature of GIC is 350 °C). A lower heating rate involves higher diffusion effects of the intercalant, which reduces the expansion ratio, in a similar manner to what observed in the process of polymer foaming [52]. 

### 3.2. Nanocomposite Characterization 

**Figure 5:**
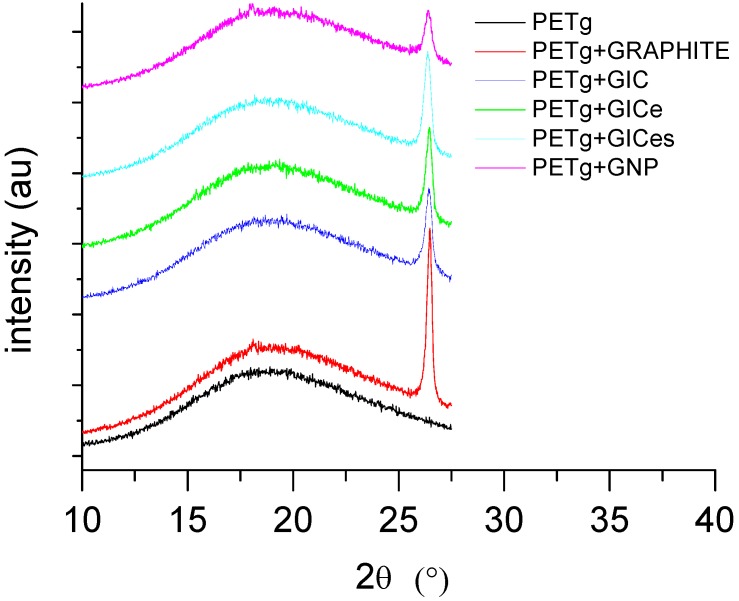
XRD traces of poly-ethylene-terephthalate (PETg) nanocomposites.

XRD analysis performed on the PETg nanocomposites is reported in Figure 5, showing that mechanical shearing, due to mixing, does not involve any significant modification of the diffraction peak of the nanofiller. This indicates that the polymer macromolecule is not able to penetrate within the very thin lamellar galleries. Therefore, the degree of dispersion of the nanofiller is mainly determined by the efficiency of exfoliation attained during the thermal treatment of GIC. 

**Figure 6:**
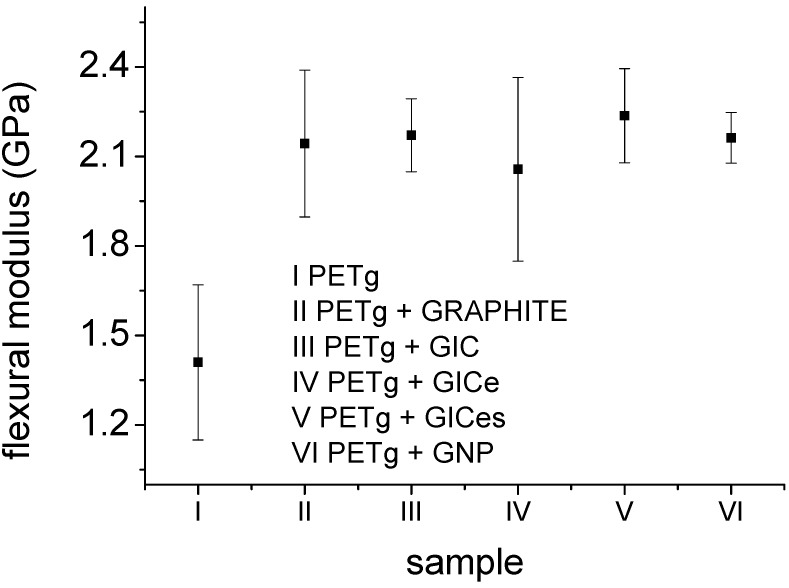
Flexural modulus for PETg and its nanocomposites.

**Figure 7:**
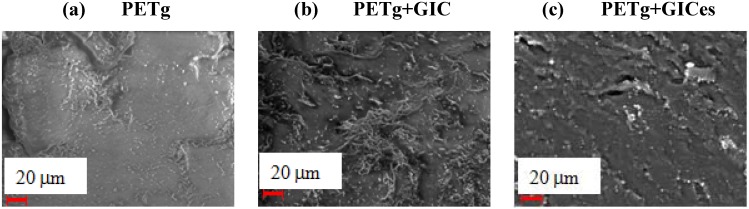
SEM micrographs of fractured surfaces.

The results for the flexural modulus obtained by mechanical tests are reported in Figure 6, showing that the addition of graphite nanoparticles involves an increase of the flexural modulus by about 30%. This is roughly equivalent to adding clay nanofillers at 8% by volume [3], which indicates the very high efficiency of graphite nanoparticles for polymer stiffening. On the other hand, no direct correlation can be observed between flexural modulus and the morphology of the nanofiller. The SEM images of fractured surfaces of PETg nanocomposites are reported in Figure 7, showing the very good adhesion between PETg matrix and nanofiller.

**Figure 8:**
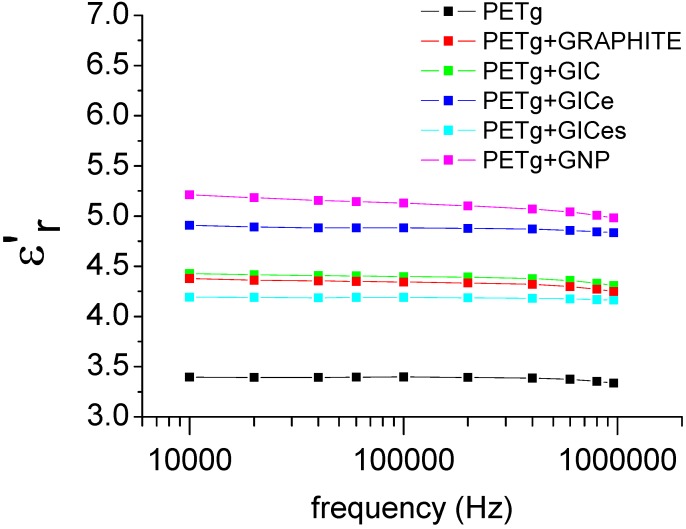
Dielectric constants of PETg nanocomposites.

The dielectric analysis results for PETg and its nanocomposites are reported in Figure 8, showing that addition of nanofillers involves a significant increase of the dielectric constant ε'_r_, due to the very high eclectic conductivity of graphite nanofillers. 

**Figure 9:**
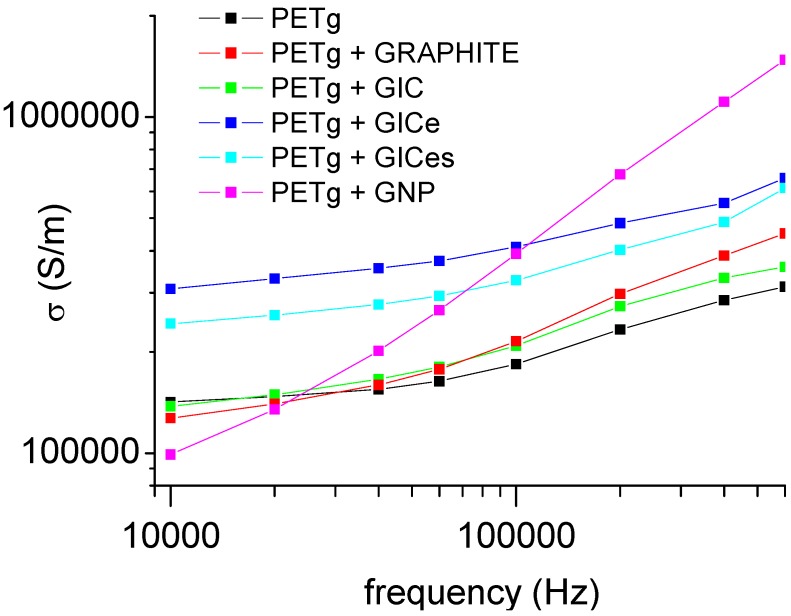
Electric conductivity of PETg nanocomposites.

The electric conductivity, obtained as *σ* = *ε''ω*, is reported in Figure 9. Even in this case, an increase of the electric conductivity is observed by the addition of graphite nanofillers. The frequency-dependent conductivity indicates that all nanocomposites can be considered insulating materials [53]. In fact, semiconductor polymer nanocomposites usually show a frequency-independent conductivity, whereby the frequency-independent conductivity is regarded as the DC conductivity.

The analysis of the dielectric constant and electric conductivity allows establishing a correlation with the degree of dispersion of nanofillers. In both cases, the increase is moderate when the GIC or graphite is added. The increase is more evident when the treated GIC, which is expected to be more finely dispersed in the matrix, is added. In particular, the electric conductivity of the nanocomposite obtained with GICe is shown to be higher than that of the nanocomposite obtained with CIGes, despite the finer dispersion of the latter. This can probably be attributed to the different structure of the two nanofillers, as reported in the SEM images. It is likely that at low nanofiller contents, the worm-like structure of the GIC, is more efficient in forming clusters of conducting nanoparticles. Therefore, by improving the degree of dispersion of the nanofiller, the dielectric properties are subjected to higher variations. 

The results from UL 94-HB burning tests are reported in Table 3, showing that the addition of the graphite nanofillers allows reducing the self-extinction time. This can be attributed to the increasing charring effect due to the presence of carbon nanofillers [4]. The best results were obtained when the GIC was added. In this case, in fact, the intumescency of the nanofiller causes, besides charring, a further reduction of the flammability. In any case, the tests showed significant dripping effects in both horizontal as well as vertical tests. 

**Table 3:** Summary of fire resistance behavior of PETg nanocomposites.

Sample	Self-estinguish	Extinction time (s)
PETg	YES	23.5
PETg + 1% GIC	YES	1
PETg + 1% GRAPHITE	YES	12
PETg + 1% GNP	YES	21.3

## 4. Conclusions 

In this work, amorphous thermoplastic matrix nanocomposites based on graphite nanoparticles were produced by melt mixing, using different types of graphite. The exfoliation of graphite intercalation compounds was studied by means of TMA analysis. Results obtained indicated that the expansion, due to evaporation of the intercalation compound, takes place by a two stage mechanism, beginning at about 170 °C and ending at about 400 °C. The expansion ratio increases with increasing heating rate and with decreasing applied pressure. The expanded GIC has a structure comparable to that of graphite nanoplatelets. XRD and SEM analysis confirmed the exfoliation, indicating the formation of a worm-like structure. The XRD analysis performed on the nanocomposites indicated that no further exfoliation takes place during melt mixing. The nanocomposites obtained by the addition of graphite nanoparticles show a significant enhancement of properties. In particular, all the nanocomposites show an increase of the modulus of about 30% compared to neat matrix, with only 1% content of nanofiller. The flexural modulus of the nanocomposite does not show any correlation to the degree of dispersion of the nanofiller, and in any case, the observed difference between samples was much lower than the error bars of the tests. Also, the nanocomposites obtained with graphite nanofillers showed a remarkable improvement of fire resistance. The effect was particularly evident for nanocomposites obtained with GIC, in which intumescency caused a reduction of the self-extinction time. On the other hand, PETg nanocomposite also showed an improvement of electrical conductivity. In this case, it was possible to establish a direct correlation between the improvement of electrical conductivity and nanofiller dispersion. Nanocomposite obtained with coarser particles (graphite and GIC) showed a moderate increase of electrical conductivity, whereas nanocomposites produced with finely dispersed particles (GICe and GICes) showed a significant improvement of the electrical conductivity. 
